# Predicting translational deformity following opening-wedge osteotomy for lower limb realignment

**DOI:** 10.1007/s11751-015-0232-4

**Published:** 2015-09-22

**Authors:** Richard C. Barksfield, Fergal P. Monsell

**Affiliations:** Bristol Royal Hospital for Children, Paul O’Gorman Building, Upper Maudlin Street, Bristol, BS2 8BJ UK

**Keywords:** Opening-wedge osteotomy, Deformity analysis, Osteotomy rules, Translational deformity, Limb realignment, Obligatory translation

## Abstract

An opening-wedge osteotomy is well recognised for the management of limb deformity and requires an understanding of the principles of geometry. Translation at the osteotomy is needed when the osteotomy is performed away from the centre of rotation of angulation (CORA), but the amount of translation varies with the distance from the CORA. This translation enables proximal and distal axes on either side of the proposed osteotomy to realign. We have developed two experimental models to establish whether the amount of translation required (based on the translation deformity created) can be predicted based upon simple trigonometry. A predictive algorithm was derived where translational deformity was predicted as 2(tan *α* × *d*), where *α* represents 50 % of the desired angular correction, and *d* is the distance of the desired osteotomy site from the CORA. A simulated model was developed using TraumaCad online digital software suite (Brainlab AG, Germany). Osteotomies were simulated in the distal femur, proximal tibia and distal tibia for nine sets of lower limb scanograms at incremental distances from the CORA and the resulting translational deformity recorded. There was strong correlation between the distance of the osteotomy from the CORA and simulated translation deformity for distal femoral deformities (correlation coefficient 0.99, *p* < 0.0001), proximal tibial deformities (correlation coefficient 0.93–0.99, *p* < 0.0001) and distal tibial deformities (correlation coefficient 0.99, *p* < 0.0001). There was excellent agreement between the predictive algorithm and simulated translational deformity for all nine simulations (correlation coefficient 0.93–0.99, *p* < 0.0001). Translational deformity following corrective osteotomy for lower limb deformity can be anticipated and predicted based upon the angular correction and the distance between the planned osteotomy site and the CORA.

## Introduction

The management of lower limb deformity by corrective osteotomy is complex and requires a thorough understanding of the deformity analysis. The advent of computerised imaging systems and the availability of digital templating software have simplified the pre-operative planning process but still require an appreciation of the limitations imposed by simple mathematical principles.

Paley et al. [1] stressed the importance of the centre of rotation of angulation (CORA) in planning corrective osteotomies and highlighted that where a corrective osteotomy was performed at an alternative position to the CORA, translation at the osteotomy would be needed if the limb axis was to be correctly realigned. This phenomenon, described as obligatory translation, follows the principles of mechanics in deformity correction [2].

It is not always possible to perform a corrective osteotomy at the CORA due to a number of factors: severe juxta-articular deformities or previous instrumentation may render bone stock insufficient to provide adequate fixation, and soft tissue scarring or tenuous skin coverage may prevent access to the osteotomy site altogether [3].

Whilst the need for obligatory translation in an osteotomy away from the CORA has been appreciated, the amount required for a size of angular correction plus distance from the CORA has yet to be calculated. We designed a study to establish the relationship between translational malalignment occurring following osteotomy using a digital image model. Our null hypothesis was that there was no correlation between the site of corrective osteotomy and the resultant translational deformity based on our suggested trigonometric method.

## Materials and methods

The study looked at a single uniplanar corrective osteotomy to restore the anatomical alignment of the affected bone in the coronal plane. We assumed obligatory translation would apply equally to osteotomies at any level within the lower limb but sought to confirm this by analysis of osteotomies at three different levels: the distal femoral metaphysis, the proximal tibial metaphysis and the distal tibial metaphysis.

### Derivation of predictive algorithm

**Fig. 1:**
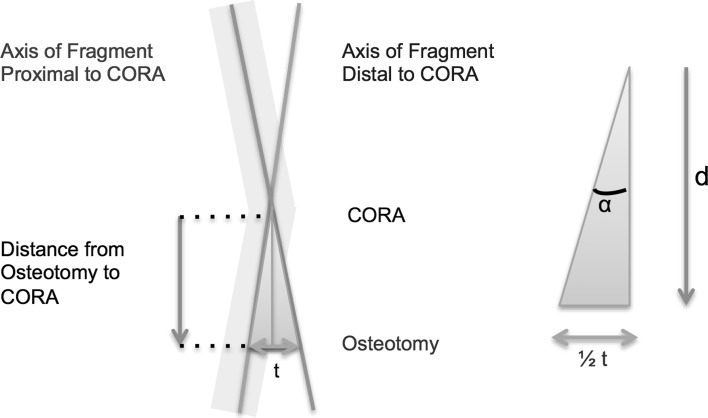
Derivation of predictive algorithm. The anatomical axis of the proximal and distal fragments intersects at the centre of rotation of angulation (CORA). Where the osteotomy is performed away from the CORA, the translation encountered is 2(tan *α* × *d*)

**Fig. 2:**
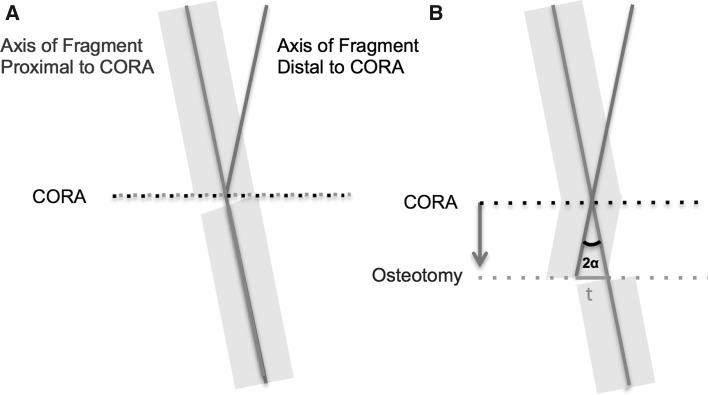
Demonstration of where an opening-wedge osteotomy is performed at the CORA, no translation is necessary in order to correct the axis of deformity (**a**). Where the osteotomy is at in an alternative position to the CORA, translation is obligatory if the axis of deformity is to be realigned (**b**)

The predictive algorithm was developed using a simplified model comprising of a CORA at the intersection of a proximal anatomical axis and a distal anatomical axis. The angle subtended at the intersection of these axes was the angular correction needed. The predicted translation was calculated by resolving the model into two identical right-angled triangles. In this way, the anticipated translation would be 2(tan *α* × *d*), where *α* represents 50 % of the desired angular correction, and *d* is the distance of the desired osteotomy site from the CORA (Figs. 1, 2).

#### Digital image simulation

**Fig. 3:**
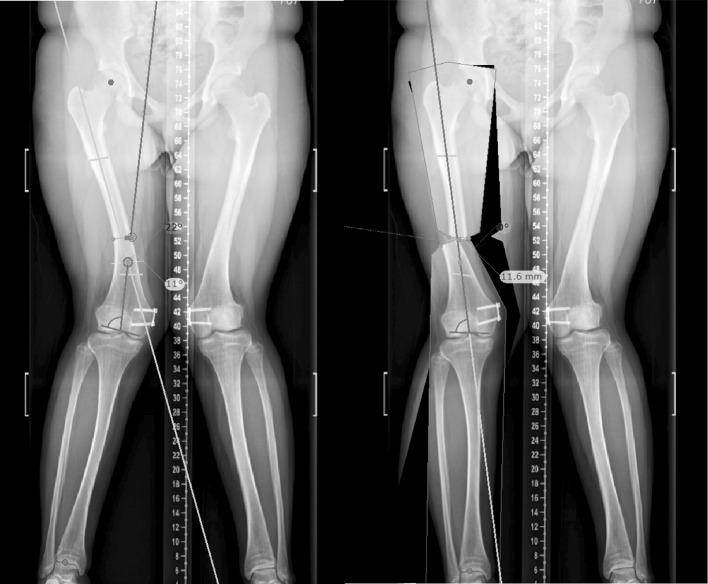
Demonstration of the use of the deformity templating software for a typical distal femoral deformity simulation. Note the translation that occurs where the osteotomy is performed proximal to the CORA

Experimental data were simulated using the TraumaCad online digital software suite (Brainlab AG, Germany). Anonymised lower limb scanograms were uploaded and calibrated using the inbuilt “Deformity Correction model”. A lower limb deformity analysis was performed for each scanogram, and a CORA derived for correcting each deformity. A corrective osteotomy and limb realignment were then simulated at the level of the CORA; this was repeated at intervals of 5 mm proceeding proximally from the distal femoral and distal tibial metaphyses, and proceeding distally from the proximal tibial metaphysis (Fig. 3). For each simulation, a series of measurements was performed that included the angular correction, the distance to CORA, the measured translation and the percentage translation.

The predicted values for each angular deformity were calculated for each level of osteotomy and correlated with the data generated from the simulated radiological data. Data were analysed using GraphPad Online Statistics Calculator [4] and STATA (StataCorp LP, TX, USA). Agreement between simulated and predicted models was then assessed using Bland and Altman limits of agreement and Lin’s concordance correlation coefficient. This was an important step in determining true agreement between the experimental models rather than a linear association that may be affected by systematic bias.

## Results

### Digital simulation

Simulated data were collected for nine experimental models that comprised of three distal femoral, three proximal tibial and three distal tibial deformities. The mean angular correction simulated was 23.0° and this ranged from 9.8° to 40.4°.

### Distal femur

**Fig. 4:**
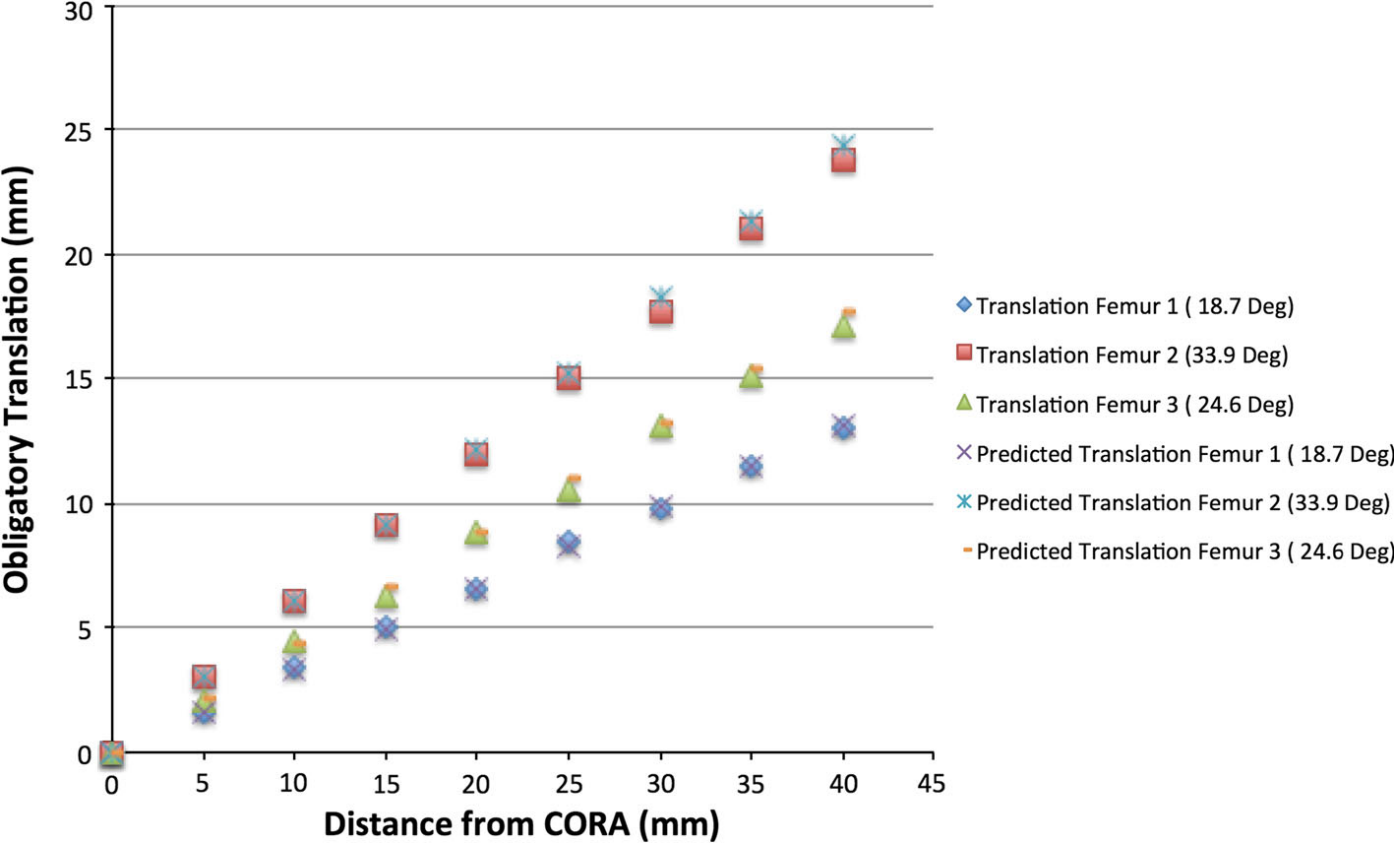
Simulated and predicted data for correction of distal femoral deformities. There was strong correlation between translational deformity and increasing distance of the osteotomy from the CORA in the simulated model. In addition, there was strong correlation between predicted translation and the translation measured during simulation

Data for the distal femoral deformities are presented in Fig. 4. The correlation coefficient between distance of the osteotomy from the CORA and translational deformities produced was 0.99 (*p* < 0.0001) for all femurs studied.

### Proximal tibia

**Fig. 5:**
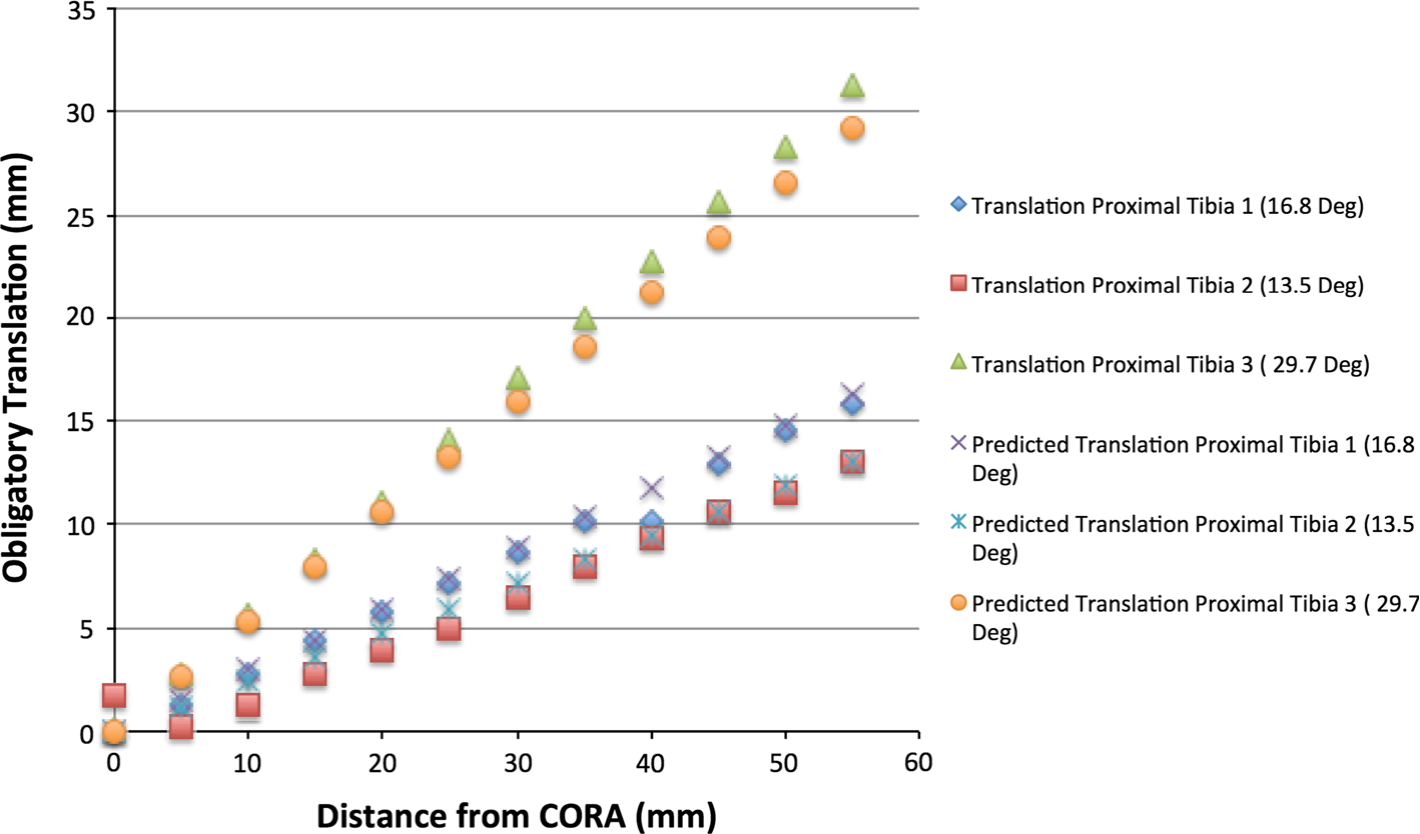
Simulated and predicted data for correction of proximal tibial deformities. There was strong correlation between translational deformity and increasing distance of the osteotomy from the CORA in the simulated model. Note that translation for tibia 2 decreased initially as distance from the CORA increased, but then followed the predictive model

Data for proximal tibial simulations are presented in Fig. 5. The correlation coefficient for the distance of the osteotomy to the CORA and the measured translation was 0.99 for tibia 1 (*p* < 0.0001), 0.93 for tibia 2 (*p* < 0.0001) and 0.99 for tibia 3 (*p* < 0.0001).

### Distal tibia

**Fig. 6:**
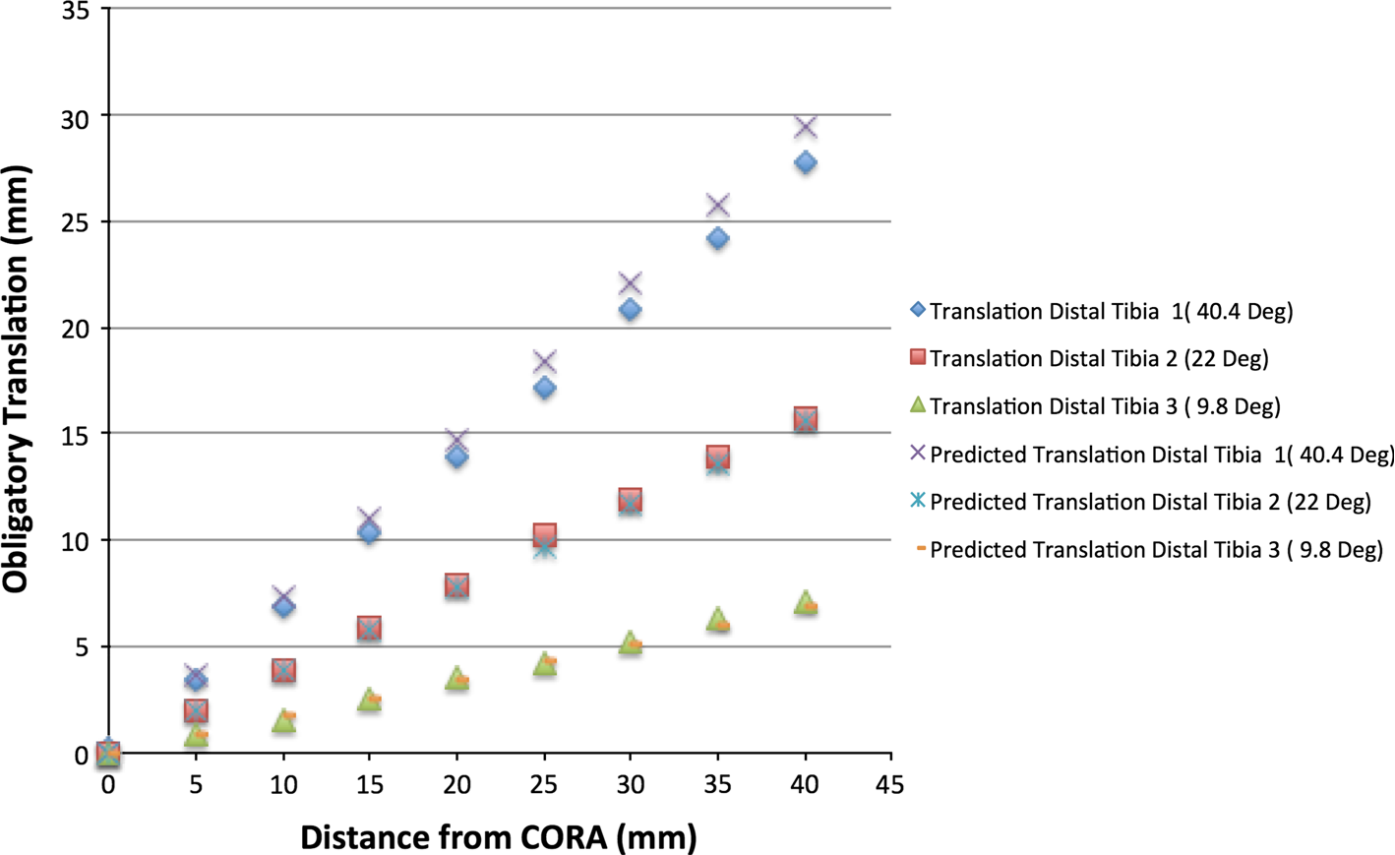
Simulated and predicted data for correction of distal tibial deformities. There was strong correlation between translational deformity and increasing distance of the osteotomy from the CORA in the simulated model. There was also a clear correlation between the predicted translational deformity and that produced by the simulated model

Simulated modelling data for distal tibial deformity corrections are presented in Fig. 6. In keeping with data from both distal femoral and proximal tibial simulations, there was a clear correlation between distance of the osteotomy to the CORA and resulting translation [correlation coefficient 0.99 (*p* < 0.0001) for all simulations].

### Agreement between experimental models

The analysis of agreement between predicted and simulated models is presented in Table 1. Substantial agreement (Lin’s concordance coefficient >0.99) was seen in eight of the nine models and even better (Lin’s concordance coefficient 0.95–0.99) in the remaining model. The Bland and Altman limits of agreement were low and include zero for all models, indicating that there was no clinically significant difference between experimental models and that no systematic bias was apparent.

**Table 1:** Lin’s concordance coefficient (LCC) and Bland and Altman limits of agreement between simulated and predicted models

Variable	Lin’s concordance coefficient			Bland and Altman agreement		
	PPMCC	LCC	95 % CI LCC	Mean difference	SD	Limits of agreement (95 %)
Proximal tibia 1	0.99	0.99	0.97–1.00	−0.31	0.497	−1.28 to 0.67
Proximal tibia 2	0.96	0.95	0.89–1.00	−0.45	0.865	−2.14 to 1.25
Proximal tibia 3	1.00	0.99	0.99–1.00	0.71	0.551	−0.37 to 1.78
Distal femur 1	1.00	1.00	0.99–1.00	0.01	0.124	−0.23 to 0.26
Distal femur 2	1.00	0.99	0.99–1.00	−0.22	0.234	−0.68 to 0.24
Distal femur 3	1.00	0.99	0.99–1.00	−0.21	0.220	−0.64 to 0.22
Distal tibia 1	1.00	0.99	0.99–1.00	−0.86	0.613	−2.06 to 0.34
Distal tibia 2	1.00	0.99	0.99–1.00	0.16	0.156	−0.15 to 0.46
Distal tibia 3	0.99	0.99	0.99–1.00	0.04	0.160	−0.28 to 0.35

## Discussion

**Fig. 7:**
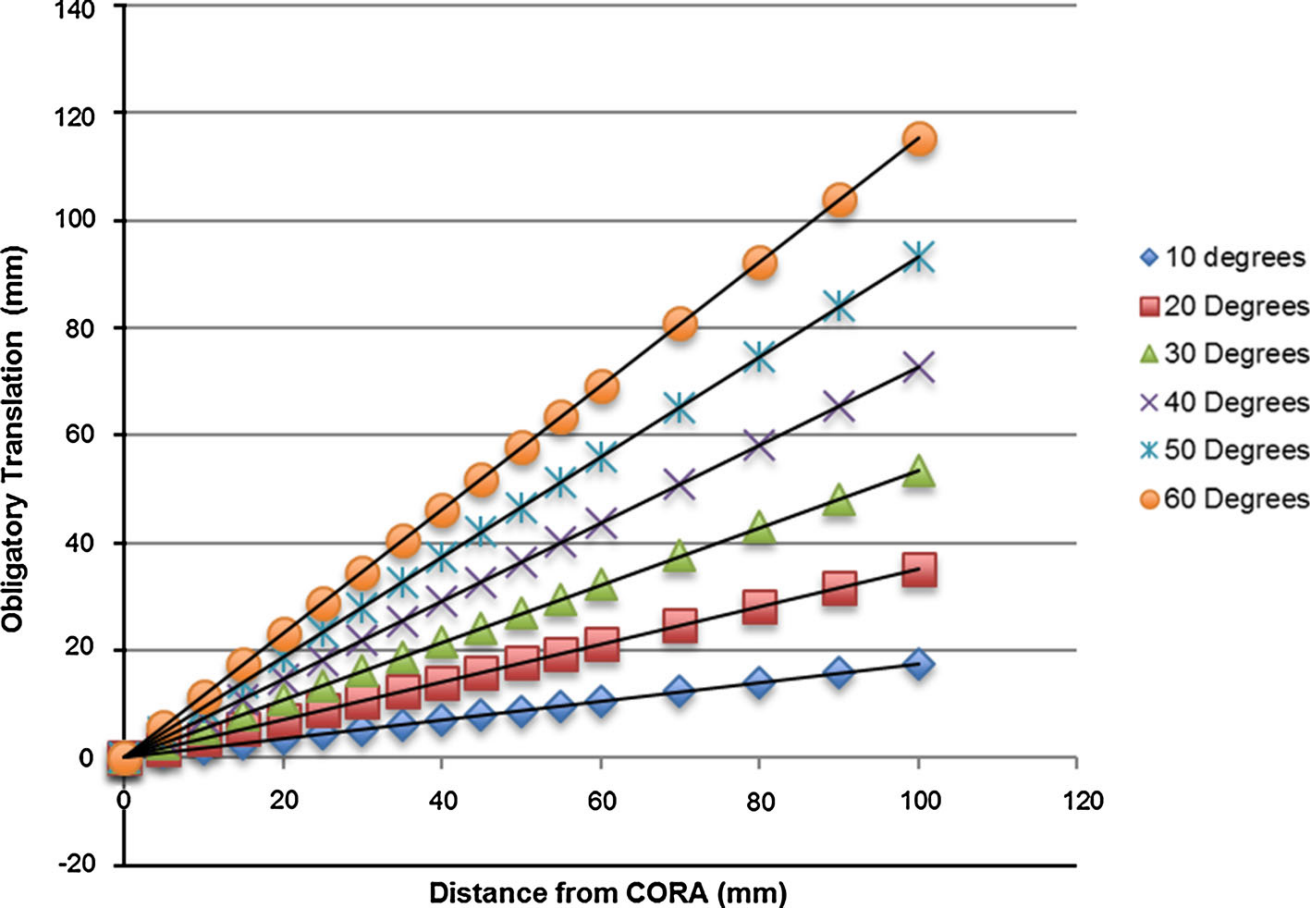
Chart demonstrating the predicted translational deformity for opening-wedge osteotomy for a range of angular corrections and distances from the CORA

Translation following corrective osteotomy for lower limb deformity can be anticipated and predicted based upon the angular correction and the relationship between the planned osteotomy site and the CORA. In most cases, significant translational deformity can be avoided by an appreciation of the site of the CORA and execution of the osteotomy at this level. There are, however, occasions where optimal placement of the osteotomy site is not possible either due to soft tissue considerations and previous instrumentation, or periarticular deformity in which fixation may become tenuous in the resulting bone fragments [5]. Under these circumstances, it is useful to have an appreciation of the obligatory translation that will result, and we have therefore developed a predictive model to estimate this in most cases (Fig. 7; Table 1).

We have demonstrated a strong correlation between translational deformity and osteotomy site, and excellent agreement between predictive and simulated models. This is not surprising when considering that the predictive model was derived from a simplified mathematical model and these are the laws upon which the deformity correction software is based. We recognise that both models may be an oversimplification, particularly when considering the constraints of the soft tissue envelope on the angle of correction achievable and the actual translation observed at the osteotomy site (Table 2).

**Table 2:** Maximum distance osteotomy can be performed from CORA before 10-mm obligatory translation ensues

Angular correction (°)	Maximum distance of osteotomy to CORA (mm)
10	57
20	28
30	19
40	14
50	11
60	9

The next logical step in this process would be to compare the translation predicted by our model with the actual translation produced across a range of angular corrections “in vivo”. The practicalities of doing this are complicated. Many of our corrections are performed using circular fixators which make early radiographs difficult to interpret, and we found follow-up films after fixator removal difficult to analyse due to the overlying callus formation, poor distinction of osteotomy edges and loss of the true site of the CORA. Whilst an “in vivo” model would be preferable, we did not find early pilot data to be reliable and hence this was not included in the analysis.

With the advent of digital imaging software, a number of studies have examined the reliability of computer-assisted measurements in assessment of lower limb alignment and deformity measurement [6–8]. In the present study, the simulated modelling was performed using the TraumaCad online software suite and a single observer (RB). We have not evaluated the intra-observer and inter-observer reliability of measurements using this technique, but are aware of a previous study that has specifically examined the reliability of measurements performed using the TraumaCad software suite and found them to be reliable [9]. We therefore believe the results presented to be both relevant and reproducible.

Our null hypotheses were that there would be no relationship between the position of osteotomy with regard to the CORA and the resultant obligatory translation produced. On the basis of our results, we have rejected this hypothesis and have demonstrated not only a clear correlation between position of osteotomy and resultant translation, but also that this can be predicted by an appreciation of the geometric equation.
